# A prognosis prediction chromatin regulator signature for patients with severe asthma

**DOI:** 10.1186/s13223-023-00796-1

**Published:** 2023-05-27

**Authors:** Yaning Gao, Liang Chen, Jian Li, Zhengjun Wen

**Affiliations:** Beijing Jingmei Group General Hospital, Beijing, China

**Keywords:** Severe asthma, Chromatin regulators, Risk model, Nomogram, Epigenetics

## Abstract

Severe asthma imposes a physical and economic burden on both patients and society. As chromatin regulators (CRs) influence the progression of multiple diseases through epigenetic mechanisms, we aimed to study the role of CRs in patients with severe asthma. Transcriptome data (GSE143303) from 47 patients with severe asthma and 13 healthy participants was downloaded from the Gene Expression Omnibus database. Enrichment analysis was performed to investigate the functions of differentially expressed CRs between the groups. We identified 80 differentially expressed CRs; they were mainly enriched in histone modification, chromatin organization, and lysine degradation. A protein–protein interaction network was then constructed. The analyzed immune scores were different between sick and healthy individuals. Thus, CRs with a high correlation in the immune analysis, *SMARCC1*, *SETD2*, *KMT2B*, *and CHD8*, were used to construct a nomogram model. Finally, using online prediction tools, we determined that lanatoside C, cefepime, and methapyrilene may be potentially effective drugs in the treatment of severe asthma. The nomogram constructed using the four CRs, *SMARCC1*, *SETD2*, *KMT2B*, and *CHD8*, may be a useful tool for predicting the prognosis of patients with severe asthma. This study provided new insights into the role of CRs in severe asthma.

## Introduction

Currently, there are approximately 300 million patients with asthma worldwide [35]. As a diffuse respiratory disease, the main pathological features of asthma include airway inflammation and remodeling, which result in airflow limitation and bronchial hyperresponsiveness [17]. Standard inhalation therapy is an effective means of controlling the condition of most asthmatic patients. However, approximately 10% of the patients with asthma do not benefit from such therapies [13, 28]. Patients with asthma who require high-dose inhaled corticosteroid treatments and a second controller to prevent uncontrolled asthma attacks or who remain uncontrolled despite these treatments are considered to have severe asthma [38]. The Global Initiative for Asthma (GINA) recommends the corticosteroid, azithromycin, anti-IL4R, anti-thymic stromal lymphopoietin, long-acting muscarine anticholinergic, short-acting β-agonists, and anti-IgE antibody omalizumab for the treatment of severe asthma [31]. However, as a heterogeneous disease, severe asthma requires complex treatments [14].

Epigenetics refers to those modifications that alter chromatin and regulate gene expression without altering the underlying DNA sequence [4]. Chromatin regulators (CRs) are important factors in epigenetics; they are mainly involved in DNA methylation, histone modification, chromatin remodeling, and the production of miRNAs that affect protein concentrations in the cell [1]. Defective chromatin regulation is associated with the development of multiple diseases [11]. Corticosteroids have been among the main modalities for the treatment of asthma, but possible reasons for their inefficacy in severe asthma are the failure to recruit HDAC2/SIRT1 and the presence of oxidatively/post-translationally modified HDAC2/SIRT1 in asthmatics [29]. Epigenetic markers regulate many processes in T lymphocytes in asthma. Furthermore, the identification of DNA methylation of specific nucleotides as biomarkers of asthma has been previously reported [44]. However, an in-depth study on the role of CRs in severe asthma is important for the treatment and prognosis of this condition.

In this study, we analyzed previously determined gene expression and clinical data of patients with severe asthma and healthy individuals. We also sorted the genes encoding CRs based on information from previous literature. The comprehensive analysis of the data was expected to provide new insights into the treatment and prognosis of severe asthma.

## Materials and methods

### Data collection and preprocessing

Information from accession GSE143303 was downloaded from the Gene Expression Omnibus (GEO) database [5] of the National Center for Biotechnology Information (NCBI); it includes transcriptome data from endobronchial biopsy samples of 47 patients with severe asthma and 13 healthy participants [34]. In addition, we curated information from 870 CRs from the published literature [23]. RStudio (version 1.4.1717.0) was used to normalize the gene expression profile of GSE143303. Subsequently, expression matrices of CRs in patients with severe asthma were obtained [15]. The limma package was used to identify differentially expressed CRs according to the following criteria: |log fold change (FC)| ≥ 0.2 and P < 0.05.

Patients with severe asthma included in this study were defined as those treated for GINA step 4 or 5, requiring high doses of inhaled corticosteroids (ICS) and a second “controller” after which the condition remained uncontrolled and either had persistent symptoms and/or worsened. Specifically, the high dose of ICS refers to > 500 µg fluticasone or equivalent per day [31]; Sánchez-Ovando et al. [32]. Clinical characteristics of patients with severe asthma and healthy controls have been presented in a previously published article (Sánchez‐Ovando et al. 2021).

### Enrichment analysis of differentially expressed CRs

To understand the potential function of the differentially expressed CRs, Gene Ontology (GO) analysis, including the GO terms molecular function (MF), biological process (BP), and cellular component (CC), was performed using the aclusterProfiler R package. A Kyoto Encyclopedia of Genes and Genomes (KEGG) pathway-based analysis was also performed. Enrichment results were visualized using the enrichplot package.

### Construction a protein to protein interaction (PPI) network

Using STRING, a PPI network of differentially expressed CRs was constructed [37]. The top 10 hub genes were identified by applying the MCC algorithm of the cytoHubba plugin of Cytoscape.

### Immune function analysis

The ssGSEA, GSEABase, and GSVA algorithms were used to evaluate the infiltration of 16 different immune cell types and 13 immune functions in samples from patients with severe asthma. The correlation between immune-infiltrating cells and immune functions was analyzed using the corrplot package in R.

In addition, the psych and ggcorrplot packages in R were used to analyze the correlations between the hub genes in the PPI network and immune-infiltrating cells and immune functions in severe asthma cases.

### Constructing a prognostic prediction model

CRs with a high correlation in immune analyses were used to construct a prognostic model of severe asthma. A receiver operating characteristic (ROC) curve was used to verify the accuracy of the model.

### Identifying potential drugs for the treatment of severe asthma

The CRs used to construct the model were entered into the Enrichr online tool [20] and the DSigDB database was used to predict the 10 most probable effective therapeutic drugs for severe asthma [43].

### Prediction of miRNAs targeting model genes

The TargetScan database (https://www.targetscan.org/vert_80/) at the Enrichr online website was used to predict the miRNAs that target those genes used for the construction of the prognostic prediction model, and a regulatory network was constructed.

## Results

### Differentially expressed CRs

**Fig. 1:**
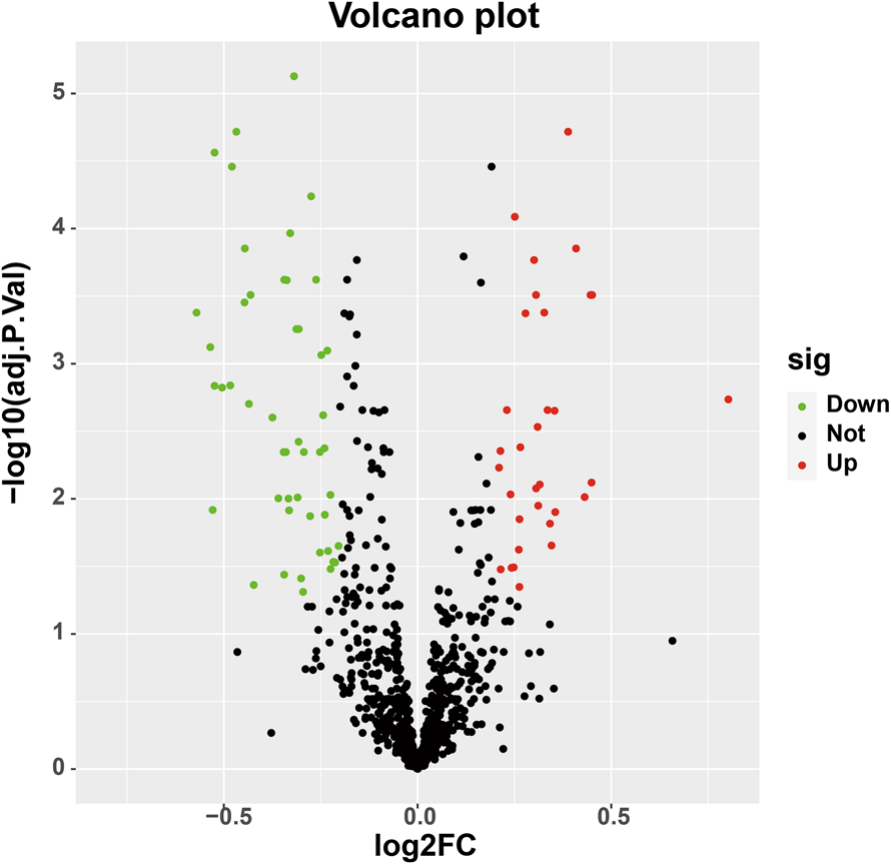
Volcano plot of differentially expressed chromatin regulators between patients with severe asthma and healthy individuals

When comparing the transcriptome data of patients with severe asthma with those of healthy individuals, we identified 80 differentially expressed CRs, including 32 upregulated and 48 downregulated genes (Fig. 1).

**Fig. 2:**
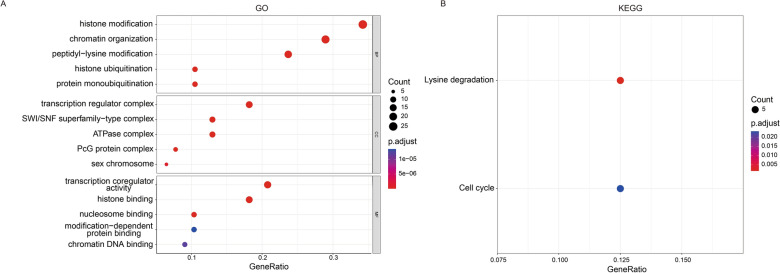
Enrichment analysis of differentially expressed chromatin regulators between patients with severe asthma and healthy individuals. **A** Gene Ontology enrichment analysis. **B** Kyoto Encyclopedia of Genes and Genomes-based pathway enrichment analysis. *MF* molecular function, *BP* biological process, *CC* cellular component

In addition, GO and KEGG analyses were performed to explore the potential functions and pathways involved in the differential expression of CRs. GO analysis indicated that these genes were mainly involved in histone modification, chromatin organization, peptidyl-lysine modification, transcription regulator complex, and transcription coregulator activity (Fig. 2A), and the enriched pathways were lysine degradation and cell cycle (Fig. 2B).

### Immune analysis of severe asthma

**Fig. 3:**
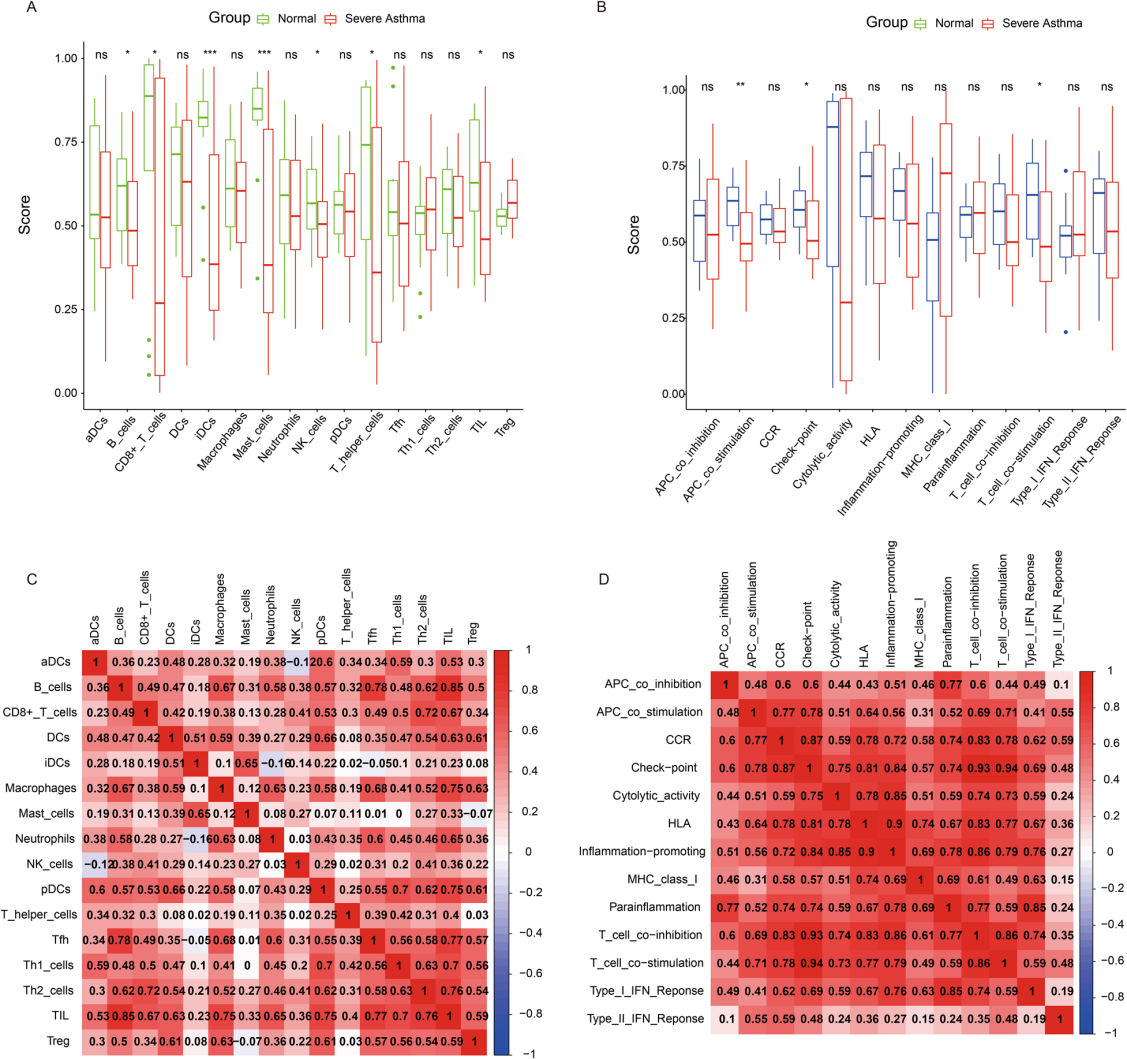
Immune analysis of patients with severe asthma and healthy subjects. **A** Immune cell scores of patients with severe asthma and healthy individuals. **B** Immune function scores of patients with severe asthma and healthy individuals. **C** Correlation analysis of immune cells. **D** Correlation analysis of immune functions

We compared the immune cell infiltration scores from patients with severe asthma and healthy controls. The results showed that the scores for B cells, CD8 + T cells, iDCs, mast cells, NK cells, T helper cells, and Th2 cells were significantly higher in healthy controls than in patients with severe asthma (Fig. 3A) (P < 0.05). In addition, the immune function scores of the two groups were compared. The results revealed that, in the healthy group, the participants’ APC co-stimulation, immune checkpoint, and T cell co-inhibition scores were significantly higher than in patients with severe asthma (Fig. 3B) (P < 0.05).

Furthermore, the correlation analysis basically showed a positive correlation between immune cells (Fig. 3C). Similar positive correlations were also observed between immune functions (Fig. 3D).

### Construction of a prognostic model

**Fig. 4:**
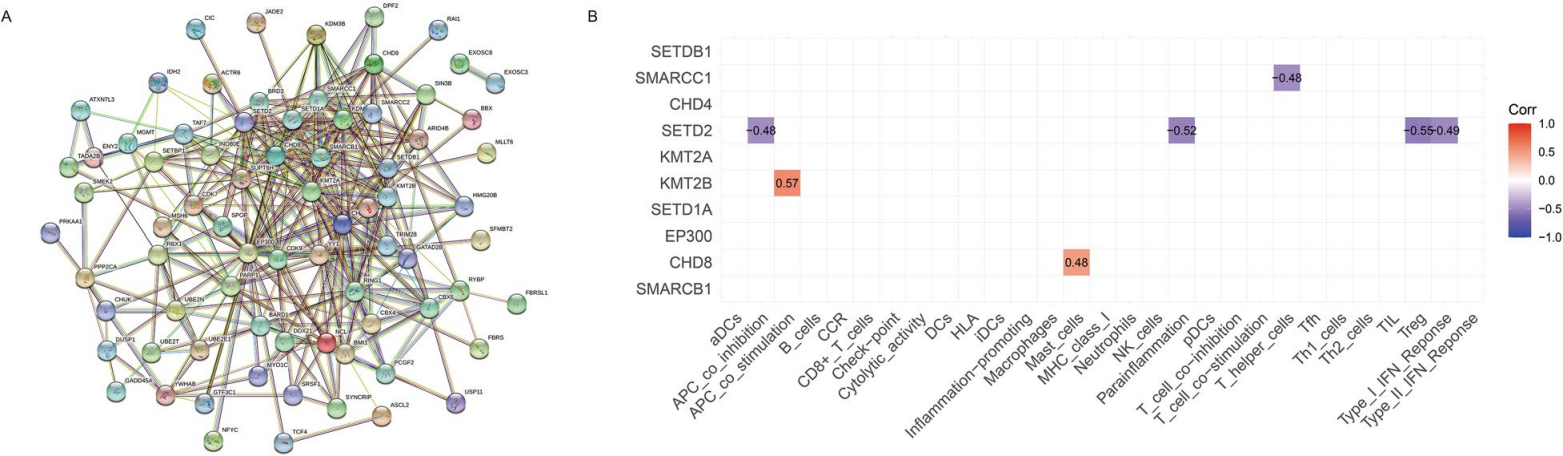
Gene interaction and immunity analyses. **A** Protein-protein interaction network of differentially expressed chromatin regulators between patients with severe asthma and healthy individuals. **B** Correlation among the ten top hub chromatin regulators in the protein-protein interaction network with immune genes and immune functions

A PPI network was constructed; it included 80 nodes and 320 edges (Fig. 4A). The ten CRs, *SMARCB1*, *CHD8*, *EP300*, *SETD1A*, *KMT2B*, *KMT2A*, *SETD2*, *CHD4*, *SMARCC1*, and *SETDB1*, in the PPI network with the highest correlations with immune genes and immune functions were further analyzed (Fig. 4B). The results showed a negative correlation between *SMARCC1* and T helper cells (r = −0.48) and a negative correlation between *SETD2* and APC co-inhibition, para-inflammation, treg, and type I IFN responses (r = −0.48, −0.52, −0.55, and − 0.49, respectively). There were also positive correlations between *KMT2B* and APC co-inhibition (r = 0.57) and between *CHD8* and mast cells (r = 0.48).

**Fig. 5:**
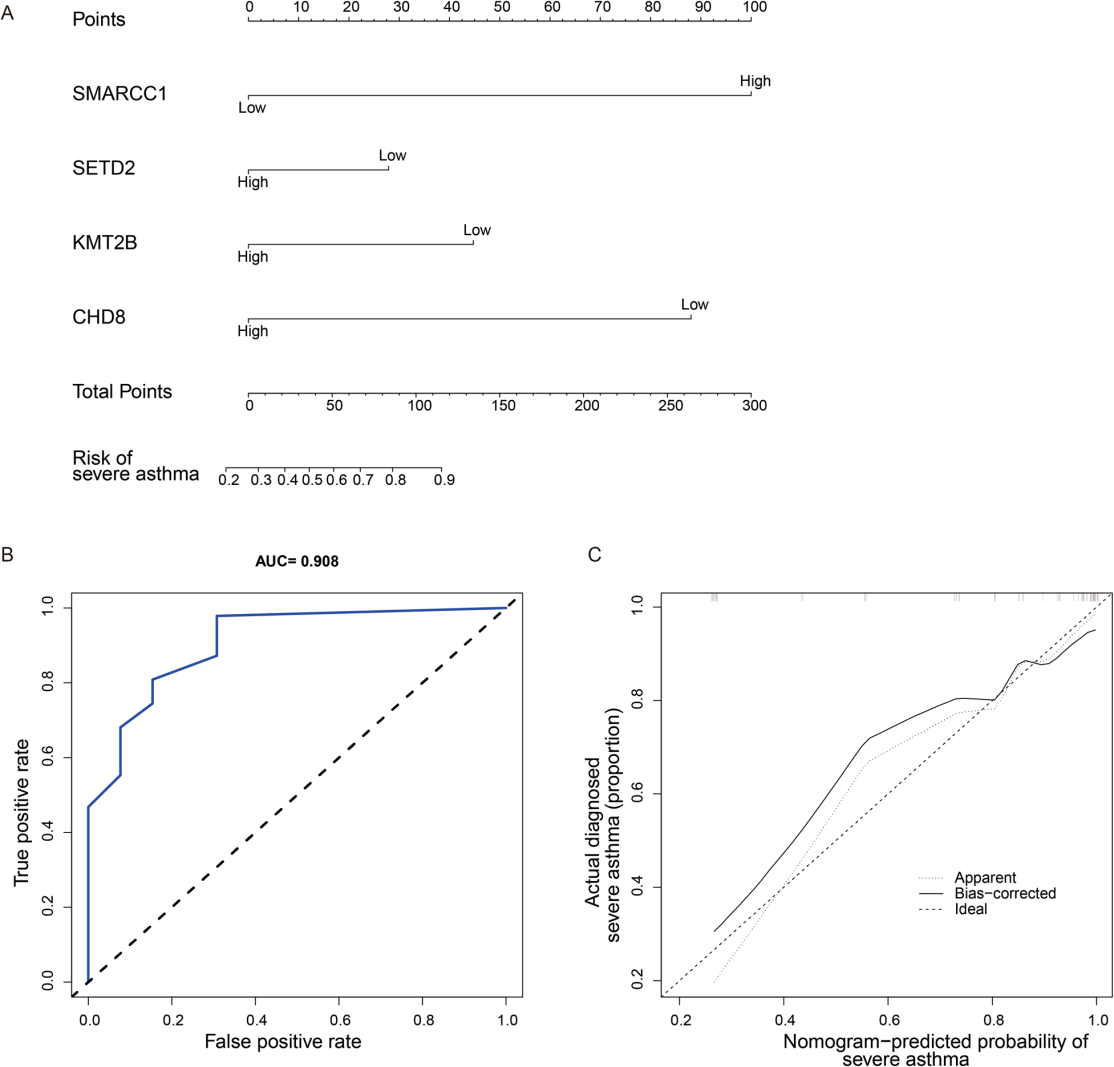
Nomogram model. **A** *SMARCC1*, *SETD2*, *KMT2B*, and *CHD8* were used to construct a nomogram model to predict the prognosis of patients with severe asthma. **B** Receiver operating characteristic curve to determine the performance of the nomogram model. **C** Calibration curve

These four genes, *SMARCC1*, *SETD2*, *KMT2B*, and *CHD8*, were used to construct a nomogram model for predicting the prognosis of patients with severe asthma (Fig. 5A). Furthermore, ROC curves showed that the AUC of this model was 0.908, indicating a good predictive performance (Fig. 5B). The calibration curves showed that the model predictions and actual values were generally consistent under ideal conditions (Fig. 5C).

### Potential drugs for treating severe asthma

DSigDB was used to predict drugs for the treatment of severe asthma, and the results showed that the ten most effective drugs would be lanatoside C, cefepime, methapyrilene, sulpiride, vitamin E, ouabain, metoclopramide, pramocaine, dirithromycin, and tamibarotene.

### Construction of an miRNA-mRNA interaction network

**Fig. 6:**
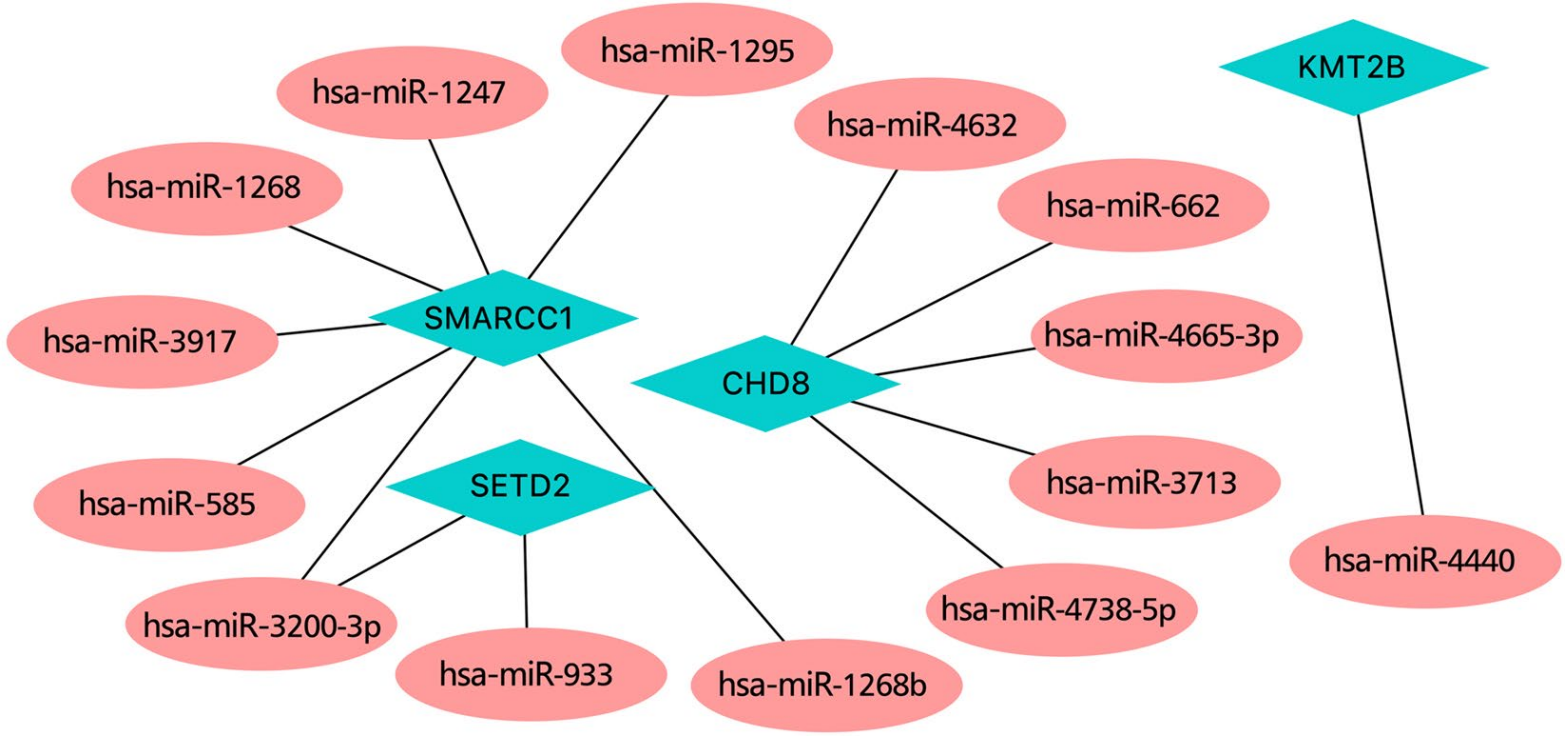
MiRNA-mRNA interaction network involving *SMARCC1*, *SETD2*, *KMT2B*, and *CHD8*.

We predicted the miRNAs upstream those genes used for the nomogram model and used them to construct an miRNA-mRNA interaction network (Fig. 6). According to our predictions, *SMARCC1* is regulated by seven miRNAs (hsa-miR-1295, hsa-miR-1247, hsa-miR-1268, hsa-miR-3917, hsa-miR-585, hsa-miR-3200-3p, and hsa-miR-1268b), *CHD8* is regulated by five (hsa-miR-4632, hsa-miR-662, hsa-miR-4465-3p, hsa-miR-3713, and hsa-miR-4738-5p), *SETD2* is regulated by two (hsa-miR-3200-3p and hsa-miR-993), and *KMT2B* is only regulated by one (hsa-miR-4440).

## Discussion

Approximately 10% of individuals with asthma are classified as having severe asthma. People with severe asthma not only have a heavy psychological and financial burden but also a high mortality rate [38]. Defects in chromatin regulation are involved in the development of various diseases [9]. To investigate the role of CRs in severe asthma, we screened for CRs that were differentially expressed between patients with severe asthma and healthy individuals. These CRs were subjected to enrichment and immunological analyses. A risk score was also constructed to assess the association between CRs and prognosis in patients with severe asthma.

The 80 differentially expressed CRs were mainly enriched in histone modification, chromatin organization, transcription regulator complex, transcription coregulator activity, lysine degradation, and cell cycle. Specialized histone modifications, as the core of chromatin control, can be removed, adjusted, or added to histone units [18]. It is known that, under specific conditions, naive CD4 + T cells are atypically activated, thus, they differentiate into a Th subpopulation cell type that drives the disease; this is a typical feature of asthma. Moreover, histone modification regulates cell lineage commitment in T cells [41], and different subtypes of T cells influence immune responses in asthma [39]. In addition, the Th17 cell lineage is subject to epigenetic plasticity through the remodeling of its chromatin structure [27]. A study found that genes associated with lysine levels may be also linked to reduced inflammation and the degradation of air pollutants, and that these genes are less abundant in asthmatics [21]. Furthermore, there are differences in serum metabolites between children with exacerbation-prone and non-exacerbation-prone asthma, with significant differences in those from the lysine pathway [7].

B cells are a major component of the adaptive immune response to house dust mite allergens. Depletion of B cells in house dust mite-sensitive mice prior to house dust mite stimulation results in decreased allergic responses [40]. Mast cells also play a role in asthma by secreting mediators with pro-inflammatory and airway constrictive effects, such as histamine and bioactive lipids [24]. However, the role of NK cells in patients with asthma remains controversial. Studies have reported that NK cells can promote the regression of inflammation by inducing eosinophil apoptosis [6]. Impaired cytotoxicity of peripheral blood NK cells has also been found in patients with severe asthma, suggesting an impaired ability to manage severe asthmatic inflammation [3, 10]. This study revealed differences in multiple immune cells between patients with severe asthma and healthy individuals.

To further investigate the relationship between CRs and prognosis in patients with severe asthma, we constructed a prognostic prediction model using the four identified key CRs: *SMARCC1*, *CHD8*, *SETD2*, and *KMT2B*. The model showed a good predictive performance for prognosis. It is known that the association of the SWI/SNF chromatin remodeling complex with cell cycle checkpoint genes controls cell proliferation and that SMARCC1 is an important member of the SWI/SNF complex; SMARCC1 also plays an important role in development [8]. The SWI/SNF complex has been observed in chronic rhinosinusitis, and it is possibly involved in the pathophysiology of the disease [19]. Patients with high blood eosinophil counts had lower levels of expression of the BAF155 protein, whereas patients with high histopathological eosinophil counts had lower expression of all SWI/SNF subunits [19].

SETD2 is a histone modifier responsible for the trimethylation of lysine 36 of histone H3 (H3K36) [22]. Air pollution has been linked to several lung diseases, and particulate matter of 10 μm in diameter (PM10) induces aneuploidy and leads to the generation of chromosomal instability in A549 cells by downregulating SETD2 [33]. Our model also involved *KMT2B*, which encodes an enzyme involved in histone H3 lysine 4 (H3K4) methylation [26], and *CHD8*, which encodes for a member of the chromodomain-helicase-DNA binding protein family that has been reported to play a role in transcriptional regulation, epigenetic remodeling, and other processes [25].

To further investigate the role of model genes in severe asthma, we constructed an miRNA-mRNA regulatory network. The results showed that all genes, except *KMT2B*, were regulated by multiple miRNAs, suggesting complex regulatory relationships. In addition, predicted drugs also provide a basis for the future treatment of severe asthma.

We found that differentially expressed CRs are mainly involved in cell cycle pathways. Severe asthma is characterized by proliferation of airway smooth muscle (ASM) [12]. Stimulation, including growth factors and extracellular proteins, regulates mitosis, which in turn induces ASM cell proliferation [42]. The patients included in this study were partially treated with ICS or oral corticosteroids (OCS) in a previous study (Sánchez‐Ovando et al., 2021). The anti-asthmatic approach described above is an effective inhibitor of ASM cell proliferation. Corticosteroids inhibit the signaling pathways of cell cycle progression (Ammit and Panettieri Jr, 2001). Differentially expressed CRs have also been found to be involved in lysine degradation. Lysine residues can increase pro-inflammatory factor activity and affect collagen synthesis. Thus, lysine degradation can modulate airway inflammation and airway remodeling, which are key pathogenic features of asthma [21]. Drugs that target lysine may be important in the treatment of severe asthma.

CRs control chromatin structure and function by catalyzing and binding histone modifications and are regulators of epigenetics [30]. Asthma patients were found to have enhanced histone acetyltransferases activity and reduced histone deacetylases activity. These modifications may lead to increased expression of genes associated with the inflammatory response profile of asthma [16]. Another study related to differentially expressed chromatin-modifying enzymes found that cigarette smoke differentially affected the expression of epigenetic regulators in patients with chronic obstructive pulmonary disease, further regulating the expression of target genes [36]. This study is the first to investigate the role of differentially expressed CRs in severe asthma, which may provide new targets for the treatment of asthma in the future.

This study had some limitations. First, the sample size may not be sufficiently representative. Second, the results were not experimentally validated. Moreover, multiple prospective studies are still needed.

In conclusion, this study constructed a risk model with good predictive performance by screening for differentially expressed CRs between subjects with severe asthma and healthy individuals and by selecting hub CRs among them. The results of this study provide new insights into the mechanisms underlying CRs in severe asthma.

## Data Availability

The datasets GSE143303 for this study can be found in the GEO database Home-GEO-NCBI (https://www.ncbi.nlm.nih.gov/geo/) .Data openly available in a public repository.
